# Book Reviews

**Published:** 1894-07

**Authors:** 


### Book Reviews.

An American Text-Book of the Diseases of Children, including special chapters on Essential Surgical Subjects; Diseases of the Eye, Ear, Nose and Throat; Diseases of the Skin; and on the Diet, Hygiene and General Management of Children. By American teachers. Edited by Louis Starr, M. D., Physician to the Children's Hospital, and Consulting Pediatrist to the Maternity Hospital, Philadelphia; Late Clinical Professor of Diseases of Children in the Hospital of the University of Pennsylvania; Member of the Association of American Physicians, and of the American Pediatric Society; Fellow of the College of Physicians of Philadelphia, etc. Assisted by Thompson S. Westcott, M. D., Attending Physician to the Dispensary for Diseases of Children, Hospital of the University of Pennsylvania; Physician to Out-Patient Department, Episcopal Hospital; Fellow of the College of Physicians of Philadelphia. Royal 8vo, pp. xiv.—1,190. Illustrated with wood-cuts and twenty-eight half-tone and colored plates. Sold by subscription only. Price, cloth, $7.00; sheep, $8.00; half Russia, $9.00. Philadelphia: W. B. Saunders, 925 Walnut street. 1894.

There is no department of medicine in the successful practice of which greater tact, skill, ingenuity and acumen are required, than in the branch now generally known under the name of pediatrics. During infancy and childhood many diseases are contracted or developed, which, if neglected, lay the foundation for early decay or later years of chronic invalidism. On the other hand, if these diseases are arrested promptly, through the agency of a skilful physician or an accomplished nurse, not only is future suffering avoided, but children become self-supporting who would otherwise be a tax on the public treasury. Physicians and nurses are often jointly needed to contribute to this success, and we regard the training of a nurse for the care of children who are ill as much of a specialty as is the practice of pediatrics by the physician.

The appearance of this treatise has impelled us to offer the foregoing as a prelude to what we may say in regard to the work itself. Indeed, it embraces nearly all we shall say on the subject, for it is a book that is beyond the requirements of a technical or analytical review. The editor has endeavored to cover nearly the entire field of pediatrics in a single volume. Inasmuch as the work is of an encyclopedic character, we think this a mistake. It is difficult to condense some of the subjects sufficiently to meet the limitations of such a work, and at the same time make the treatment of them sufficiently exhaustive to meet adequately the requirements of the present age. This would seem to be especially the case with such subjects as tuberculosis and hereditary syphilis.

Moreover, the book is now a cumbersome one to handle, which difficulty might have been avoided by breaking it up into two or three volumes, which would also have permitted a greater elaboration of some of the subjects. Notwithstanding all this, we feel impelled to say that Starr has produced by far the best treatise on diseases of children that has appeared in the English language. He has carefully considered such practical points as etiology, symptomatology, diagnosis and treatment, including feeding, hygiene, therapeutics and the prevention of disease, while avoiding as far as possible the insertion of references to journals or authorities. This increases the interest in the work of those for whom it was especially prepared—namely, physicians in active professional practice.

A large staff of contributors has been employed, who are, for the most part, either distinguished in the department of pediatrics, or well known as authors on the subjects upon which they write. They, too, are well distributed, geographically speaking, throughout the country, thus giving the treatise a breadth that should characterize such a work, making it a national expression of professional opinion.

The illustrations are of a character to merit approbation. They embrace almost every kind of reproductive art and are a great help to a better understanding of the text in the majority of instances. Most of them are sufficiently new to attract attention, and this is especially the case with the photographic plates. We are of the opinion that greater attention should be paid to the illustration of medical books than is frequently the case. A well-executed drawing or especially a good photographic reproduction arrests the attention of the eye at once, even of the busiest physician, and often leads him to a closer examination of the text.

One of the most important parts of a text-book is the index. This one contains an elaborately prepared index covering forty pages in three columns, making it easy of reference and greatly enhancing its value.

In conclusion we commend this work to the careful examination of every family physician. It ought to be found on his bookshelves, and it ought also to be frequently consulted.

MEDICAL JURISPRUDENCE, Forensic Medicine and Toxicology. By R. A. WITTHAUS, A. M., M. D., Professor of Chemistry, Physics and Hygiene in the University of the City of New York, etc., and Tracy C. Becker, A. B., LL. B., Counselor-at-Law and Professor of Criminal Law and Medical Jurisprudence in the University of Buffalo. In four volumes. Volume I. Large 8vo, 845 pages, illustrated with wood-cuts and two lithographic plates in colors. Price, in muslin, $5.00 ; in brown sheep and in law style, $6.00 per volume. Sold by subscription only. New York: William Wood & Company. 1894.

This treatise has been heralded by advance press notices, setting forth its scope and purpose in detail. An examination of the first volume indicates that the promises made by the editors and publishers were not extravagant. The legal relations of physicians and surgeons, including their acquirement of the right to practise medicine and surgery, their legal duties and obligations, their right to compensation, their privileges and duties when summoned as witnesses in courts of justice and their liability for malpractice forms the first section of the book, and is written by Mr. Tracy C. Becker, the well-known counselor-at-law of Buffalo, one of the editors-in-chief. In view of the action by a number of the states providing for separate license to practise medicine, it is interesting to read in Mr. Becker's opening chapter that the importance of prescribing certain educational qualifications for physicians was recognized as early as the year 1422, when, during the reign of Henry the V. in England, an act of parliament forbade anyone, under a penalty of both fine and imprisonment, from practising medicine as a means of livelihood, "unless he hath studied it in some university and is at least a bachellor of science."

The legal status of physicians is too little understood by themselves, and much information not to be found elsewhere may be gleaned by a study of the five chapters that Mr. Becker devotes to the consideration of this subject.

The next section, relating to the law of evidence concerning confidential communications between physician and patient, is treated of by Charles A. Boston, of the New York City bar. It is important that every physician called to the witness stand shall be familiar with the details given in this section.

Beginning with page 137 and ending with page 291, is a section containing a synopsis of the existing statutes which regulate the acquirement of the right to practise medicine and surgery in the United States, Great Britain and Ireland, and the Canadian Provinces. The value of this treatise as a work of reference is very great, but it is worth the price alone to possess it for the examination of these pages, eighty-seven of which are devoted to laws of the United States, and the remaining sixty-seven to those of the other countries named. This section closes the division of medical jurisprudence.

The division of forensic medicine begins here. It is subdivided into three heads: first, thanatological, including those branches in which the subject of inquiry is a dead body; second, bio-thanatological, relating to questions concerning both dead bodies and living persons; third, biological, relating to living persons. In this volume the thanatological branch of the subject is considered and begins with the legal status of the dead body, the disposal and obligation to dispose of the same, how and by whom it may be exhumed or removed, the rights of relatives and accused persons, and includes an appendix containing a synopsis of the statutes of the different United States and territories concerning the same. This section is also written by Mr. Tracy C. Becker, co-editor-in-chief.

The next sections are as follows: The powers and duties of coroners, medico-legal autopsies, personal identity, determination of the time of death, medico-legal consideration of wounds, medico-legal consideration of gunshot wounds, death by cold and heat, medico-legal relations of electricity, medico-legal consideration of death by mechanical suffocation, death by submersion or drowning, and death from starvation. Each one of these sections is written by an author specially fitted to prepare the same, and who may be deemed an expert either in law, medicine or surgery.

---

A TEXT-BOOK OF THE DISEASES OF WOMEN. By HENRY J. GARRIGUES, A. M., M. D., Professor of Obstetrics in the New York Post-graduate Medical School and Hospital; Gynecologist to St. Mark's Hospital in New York City; Gynecologist to the German Dispensary in the City of New York; Consulting Obstetrician to the New York Infant Asylum; Obstetric Surgeon to the New York Maternity Hospital; Fellow of the American Gynecological Society; Fellow of the New York Academy of Medicine; President of the German Medical Society of the City of New York, etc. Octavo, pp. 690, containing 310 engravings and colored plates. Price, cloth, $4.00 net; sheep, $5.00 net. Philadelphia: W. B. Saunders, 925 Walnut street. 1894.

We approach examination of this work with much trepidity. There are many reasons why the appearance of another treatise on diseases of women at this time is not recognized as a necessity.

Nevertheless, the ability of the present author challenges everywhere admiration and respect and a patient investigation of his writings.

Garrigues offers among the reasons for writing this book: First, that he had in view those physicians who, without previous hospital training attend a post-graduate school to learn gynecology; second, those who would like to go to such an institution, but who find it impossible to leave their practice; third, general practitioners in country places; fourth, under-graduates studying in medical colleges. As these groups make up a large proportion of medical men, it is quite probable if the book reaches them all that the first edition will promptly be exhausted.

The author divides his treatise into two general parts, which he calls general division and special division. Under the first he groups the anatomy, physiology and development of the female genitals and pelvic organs, etiology in general, examination in general, treatment in general, abnormal menstruation, and metrorrhagia and leucorrhea. In the special division may be found grouped diseases of the vulva, diseases of the perineum, diseases of the vagina, diseases of the uterus, diseases of the Fallopian tubes, diseases of the ovaries, diseases of the pelvis and in an appendix sterility.

This classification is quite novel and appeals to the practical man. Indeed, Garrigues asserts that his aim has been to write a practical work and he has done so, hence has omitted the historical development of gynecology as well as reports of special cases. In the first place we desire to speak a friendly word for the author's good judgment in omitting diphthongs wherever practicable. We hope soon to see an end of such spelling as *gynæcology*, *leucorrhœa*, *dysmenorrhœa*, and the like. The illustrations, too, deserve a mead of praise, though in many instances they are old friends borrowed from familiar sources. The photograph, too, might have been used to greater advantage in depicting many important conditions, especially those of an operative nature. We are glad to see a proper table (Daggett's), and no chair recommended for the purposes of examination and operation. We are at a loss, however, to understand why Fitch's abdominal supporter should be given a place in such an excellent treatise. And, again, we cannot condemn too strongly the use of such a so-called uterine and abdominal supporter as is illustrated on page 443, figure 240.

Many of the illustrations in the work, however, merit the praise of originality, having come from the author's own operations, dissections and microscopical examinations. The illustrations, as a whole, form a complete atlas of the embryology and anatomy of the female genitalia, and represent numerous operations and pathological conditions. The author states in his preface that he expects to be criticised for having devoted special chapters to hemorrhage and leucorrhea. We do not think he deserves criticism on that ground. Both of these conditions, though symptoms, are yet of such great moment as to stand even far beyond many diseases. Again, they are often the expression of states that need no treatment beyond the arrest of these symptoms.

An elaborate index of twenty-two pages closes this most interesting volume, which deserves the careful examination and patient reading of the profession.

---

International Clinics. A Quarterly of Clinical Lectures on Medicine, Neurology, Pediatrics, Surgery, Genito-Urinary Surgery, Gynecology, Obstetrics, Ophthalmology, Laryngology, Otology and Dermatology. By professors and lecturers in the leading medical colleges of the United States, Great Britain and Canada Edited by Judson Daland, M. D., Philadelphia, Instructor in Clinical Medicine and Lecturer on Physical Diagnosis in the University of Pennsylvania ; Assistant Physician to the University Hospital ; Physician to the Philadelphia Hospital and to the Rush Hospital for Consumptives. J. Mitchell Bruce, M. D., F. R. C. P., London, England ; Physician and Lecturer on Therapeutics at the Charing Cross Hospital. David W. Finlay, M. D.. F. R. C. P., Aberdeen, Scotland ; Professor of Practice of Medicine in the University of Aberdeen, Physician to, and Lecturer on, Clinical Medicine in the Aberdeen Royal Infirmary ; Consulting Physician to the Royal Hospital for Diseases of the Chest, London. Volume IV., Third series, 1894. Royal 8vo, pp. xii.—363. Philadelphia : J. B. Lippincott Co. 1894.

This volume of this interesting series opens with a memorial of Dr. John M. Keating, late editor-in-chief, written by his successor, Dr. Judson Daland. This touching tribute inspires one with the feeling that the world does not possess too many men like Dr. Keating and that it could illy afford to spare him.

The first lecture is on enteric fever by Sir Dyce Duckworth. The local contributors to this volume are Dr. Charles Cary, who lectures on Rupia Syphilitica, and Dr. Roswell Park, who delivers a conglomerate lecture on Chondro-Sarcoma of the Sternum, Cysto-Sarcoma of the Lower Jaw, False Ankylosis of the Shoulder, Abscess of the Temporal Bone and Basilar Meningitis, Amputation of the Forearm for Tubercular Disease of the Wrist, Recurring after Resection. Several authors of this volume also have compound titles. We think this a mistake. Single titles should be adhered to for lectures to be published in a book of this kind. This defect has occurred all through the series thus far, and we hope to see it corrected in future. The teacher loses the force and impact of his work if he fires a mitrailleuse, whereas, by a carefully prepared lecture on a single topic he leaves something substantial to the literature of medicine.

These clinics have been improved by an increase of illustrations, but there is still room for advancement in this regard.

---

A Manual of Practical Hygiene, Designed for Sanitary and Health Officers, Practitioners and Students of Medicine, By W. M. L. Coplin, M. D., Adjunct Professor of Hygiene, etc., etc., Jefferson Medical College. and D. Bevan, M. D., Instructor in Hygiene, etc., etc., Jefferson Medical College, with an introduction by H. A. Hare, M. D., Professor of Therapeutics, Materia Medica and Hygiene, in Jefferson Medical College. With 140 illustrations, many of which are in colors. Philadelphia: P. Blakiston, Son & Co.

This book is the first earnest attempt that we have seen to distribute the new wine of modern hygiene in a new bottle—a modern book—and we sincerely congratulate the authors upon their effort. Men who begin a work on hygiene by a thoughtful consideration of such topics as heredity, temperament, proclivity, immunity, idiosyncrasy, are not likely to have their work run dry, bloodless and lifeless from over much bacteriological technology. Busy students and practitioners will find in this book a happy blending of the practical matters of hygiene with a due regard for its more advanced and theoretical questions.

Unquestionably, the future is to produce many books upon this subject, and any book will require frequent revision in order to keep apace with the rapidly advancing line of progress. Our authors seem to understand this sign of the times, and have written a book along lines both ancient and modern, showing our art at its very best neither an art or a science, but artistic in purpose and scientific in method.

We notice the book as one to be commended to all students of medicine, candidates, graduates or practitioners. The questions before the hygienist are today the most practical questions of the age, and no man can afford to fall far behind the procession.

H. R.

SYLLABUS OF THE OBSTETRICAL LECTURES in the Medical Department of the University of Pennsylvania. By RICHARD C. NORRIS, M. D., Demonstrator of Obstetrics, University of Pennsylvania; Assistant Obstetrician, University Maternity, etc., etc. Third edition. Duodecimo, pp. xviii.—222. Price, $2.00 net. Philadelphia: W. B. Saunders, 925 Walnut street. 1894.

The popularity of this lecture syllabus is testified to by the fact that a third edition is so soon demanded. It is not easy to take full lecture notes unless a satisfactory guide thereto is obtained. With this book in the hands of the student it becomes much easier for him to retain the mental imprint of the work of the teacher. The improvement everywhere apparent in the teaching of the obstetric art is very gratifying. With the aid of the manikin, plates to illustrate the subject, a good lecture syllabus like the one before us and plenty of good clinical material to draw upon, a teacher ought to be able to so equip a student as to enable him to pass the State medical examining and licensing board without possibility of doubt.

The teaching of obstetrics with reference to its essential points ought to be uniformed and standardized so that there need be no misunderstanding in regard to the proper methods of conducting either a normal or an instrumental labor. By the publication of lecture syllabi the obstetric teachers in medical colleges will contribute much to this desirable end.

---

THE JOHNS HOPKINS HOSPITAL REPORTS: Vol. III., Nos. 4, 5, 6. Report in Pathology. Baltimore: The Johns Hopkins Press. 1893.

This volume of Reports is devoted to pathological investigations made at the pathological laboratories of the Johns Hopkins Hospital. Dr. Simon Flexner contributes a valuable article on The Infectious Nature of Lympho-Sarcoma, based upon the study of two cases which were treated and examined at the hospital.

Dr. Henry T. Berkley contributes an excellent article on The Cerebellar Cortex of the Dog, and, as a result of his examination of the nerve elements, concludes that the cerebellum is a sensory and not a motor organ, and that lesions of the organ, with the exception of the middle lobe, are without objective symptoms. An article on Chronic Nephritis in a Cow, by Dr. W. T. Councilman; Bacteria in Their Relation to Vegetable Tissue, by Dr. H. L. Russell; and an Analysis of 105 Cases of Heart Hypertrophy from the Autopsy Records of the Johns Hopkins Hospital conclude the report.

These Reports are the results of original investigations undertaken in the Johns Hopkins Hospital and Medical College, and give some idea of the excellent work done at this excellent school.

---

Transactions of the Southern Surgical and Gynecological Association. Volume VI. Sixth session. Held at New Orleans, La., November 14, 15 and 16, 1893. Octavo, pp. xlvii.—392. Edited by W. E. B Davis, M. D., Secretary. Published by the Association. Philadelphia: W. J. Dornan, Printer. 1894.

This volume contains thirty-two papers, including the address of the president, most of which treat of surgery of the several cavities of the body. The president's address, by Dr. Bedford Brown, of Alexandria, Va., is an able and learned exposition of the origin, objects and aims of the association. It was well received and merited the thanks of the Fellows which were unanimously given. The second paper is a memorial address in honor of Dr. Ephraim McDowell, delivered in accordance with a resolution of the association by Dr. L. S. McMurty, of Louisville. It deserves the careful reading of every gynecologist.

The discussions in this volume are up to their usual standard of excellence, but the illustrations are hardly what they should be in such a treatise. Authors would find it to their decided advantage to pay greater attention to the illustration of surgical papers. The book is carefully edited by the accomplished secretary of the association.

---

Transactions of the Medical Society of the State of North Carolina. Fortieth annual meeting, held at Raleigh, N. C., May 9, 10 and 11, 1893. Octavo, paper, pp. 154. Wilmington, N. C.: Jackson & Bell. 1893.

The annual volume of transactions of this society always possesses interest and the present one is no exception to the rule. One of the best papers in the book is a discourse upon appendicitis viewed from a personal standpoint, by Dr. J. W. Long, formerly of Randleman, now of Richmond. Dr. Long treats the subject from the surgical side of the question and in accordance with the most advanced knowledge.

We question the propriety of presenting reports on gynecology, obstetrics, surgery, etc., a plan which is still adhered to in this society. In these days of medical journals and of profuse periodical medical literature we think it would be better to abolish this plan.

The volume contains the annual reports of the State board of medical examiners and the State board of health, and ought to be more substantially bound.

---

CLINICAL LECTURES ON PEDIATRICS, delivered in the Vanderbilt Clinic during the Session of 1892–93. By A. JACOBI, M. D., Clinical Professor of the Diseases of Children in the College of Physicians and Surgeons of New York, etc., etc. (Stenographic reports) Reprinted from *Archives of Pediatrics*, Volume X., 1893. Octavo, pp. 195. New York: Bailey & Fairchild. 1893.

These lectures are delivered by one of the ablest and best-known authorities on diseases of children in the United States. They are epigrammatic in style and possess a charm that a more studied form would be deficient in. Each lecture teaches a valuable lesson on the subject or subjects of which it treats and forms most instructive reading to every physician interested in the management of diseases in children. One commendable feature of these clinical lectures is the fact that the date of the delivery of each one is given, and we assume that each gives the most advanced instruction up to the date borne at its head.

It is a great mistake for clinical teachers to omit the date of their lectures when they are published in the journals or in book form.

---

A MANUAL OF THERAPEUTICS. By A. A. STEVENS, M. D., Lecturer on Terminology and Instructor in Physical Diagnosis in the University of Pennsylvania; Demonstrator of Pathology in the Woman's Medical College, Philadelphia, etc., etc. Small 8vo, pp. 435. Price, $2.25. Philadelphia: W. B. Saunders, 925 Walnut street. 1894.

This manual is intended particularly for the use of students and has been prepared to serve as an outline of the study of modern therapeutics to be filled in by more systematic reading of larger treatises. The drugs are arranged alphabetically and to each is given its chemical formula or synonym, a brief statement of its therapeutical application and methods of its administration. Where admissible, too, its physiological action is stated and any other facts essential to an understanding of its therapeutics. A

table of doses is added and an extensive index embracing remedies and diseases closes the volume.

It will be found a handy book of reference for the medical practitioner who does not care to grope through the mass of reading in a larger text-book to get at the meat or kernel of a particular remedy.

---

ESSENTIALS OF PHYSICS. Arranged in the form of questions and answers. Prepared especially for students of medicine. By FRED. J. BROCKWAY, M. D., Assistant Demonstrator of Anatomy at the College of Physicians and Surgeons, New York. Saunders' Question-Compends, No. 22. Second edition, revised. With 155 illustrations. Duodecimo, pp. 330. Price, $1.00 net. Philadelphia: W. B. Saunders, 925 Walnut street. 1894.

The first edition of this valuable little book was soon exhausted, hence we are called upon to notice this second edition in just two years after we wrote a notice of the first. We said at that time (June, 1892, page 699,) that there are many reasons why medical students cannot devote as much time to the study of physics as they should, hence the value of such intelligent condensations of the subject as Dr. Brockway has given. He has been able to do this without doing violence to the subject or otherwise belittling its importance. It is one of the best books of the Saunders' Question-Compends Series.

---

MANUEL DE MEDECIN PRACTICIEN, la Pratique Journalière de la Chirurgie, dans Les Hopitaux de Paris, Aide-Mémoire et Formulaire de Thérapeutique Appliquée par le Professeur Paul Lefret. Paris Librairie J.—B. Baillière et Fils. 1894.

This little book is an exhibit of the daily practice of surgery in the hospitals of Paris and an aid to the memory of the formulæ of the therapeutics there in use. It is a valuable work for the purposes intended, and contains an index of authors as well as of diseases.

---

### BOOKS RECEIVED.

Treatment of Typhoid Fever. By D. D. Stuart, M. D., Lecturer on Clinical Medicine in the Jefferson Medical College of Philadelphia; Physician to the Medical Dispensary of the Episcopal Hospital, etc., etc. The Physicians' Leisure Library Series, pp. 104. Price, paper, 25 cents; cloth, 50 cents. Detroit, Mich.: George S. Davis, 1893.

The Nurse's Dictionary of Medical Terms and Nursing Treatment. Compiled for the use of nurses and containing descriptions of the principal medical and nursing terms and abbreviations, instruments, drugs, diseases, accidents, treatments, physiological names, operations, foods, appliances, etc., etc., encountered in the ward or sickroom. By Honner Morten, Author of Sketches of Hospital Life, How to Become a Nurse, etc. 32mo, pp. 139. Second edition. Price, $1.00. Philadelphia: W. B. Saunders, 925 Walnut street. 1894.

A Clinical Manual. A Guide to the Practical Examination of the Excretions, Secretions and the Blood, for the Use of Physicians and Students. By Andrew MacFarlane, A. B., M. D., Instructor in Neurology and Diseases of the Chest in the Albany Medical College; Physician to St. Peter's Hospital Out-patient Department, and Physician to Albany's Hospital for Incurables. Duodecimo, pp. xii.—139. Price, $1.25. New York: G. P. Putnam's Sons, 27 West Twenty-third street. 1894.

Transactions of the Indiana State Medical Society. Forty-fourth Annual Session, held in Indianapolis, Ind., May 11 and 12, 1893. Octavo, pp. 378. Indianapolis: Wm. B. Burford, Printer. 1893.

An Illustrated Dictionary of Medicine, Biology and Allied Sciences, including the Pronunciation, Accentuation, Derivation and Definition of the Terms used in Medicine, Anatomy, Surgery, Obstetrics, Gynecology, Therapeutics, Materia Medica, Pathology, Dermatology, Pediatrics, Ophthalmology, Otology, Laryngology, Physiology, Neurology, Histology, Toxicology, Dietetics, Legal Medicine, Psychology, Climatology, etc., etc., and the various Sciences closely related to Medicine, Bacteriology, Parasitology, Microscopy, Botany, Zoölogy, Dentistry, Pharmacy, Chemistry, Hygiene, Electricity, Veterinary Medicine, etc. By George M. Gould, A. M., M. D., Author of The Students' Medical Dictionary, 12,000 Medical Words Pronounced and Defined, The Meaning and the Method of Life; Editor of *The Medical News;* President, 1893-1894, American Academy of Medicine; one of the Ophthalmologists of the Philadelphia Hospital. Based upon recent scientific literature. Double-columned quarto, pp. xvi.—1633. Philadelphia: P. Blakiston, Son & Co., 1012 Walnut street. 1894.

Transactions of the Fifteenth Annual Meeting of the American Laryngological Association, held in the City of New York, May 22, 23 and 24, 1893. Octavo, pp. vi.—165. New York: D. Appleton & Company. 1894.

Part I., Essentials of Refraction and Diseases of the Eye. By Edward Jackson, A. M., M. D., Professor of Diseases of the Eye in the Philadelphia Polyclinic and College for Graduates in Medicine, etc., etc. Part II., Essentials of Diseases of the Nose and Throat. By E. Baldwin Gleason, M. D., Surgeon in charge of the Nose, Throat and Ear Department of the Northern Dispensary; Assistant in the Ear Department of the Philadelphia Polyclinic and College for Graduates in Medicine, etc., etc. Two volumes in one, crown 8vo, 290 pages, profusely illustrated. Price, cloth, $1; interleaved for notes, $1.25. Philadelphia: W. B. Saunders, 925 Walnut street. 1894.

Clinical Gynecology: Being a Hand-Book of Diseases Peculiar to Women. By Thomas More Madden, M. D., F. R. C. S., Ed., Obstetric

Physician and Gynecologist, Mater Misericordiæ Hospital, Dublin; Consulting Physician, Hospital for Sick Children, etc., etc. Octavo, pp. xvi.—562, with 259 illustrations. Philadelphia: J. B. Lippincott Company. 1893.

